# Barriers to the use of evidence-based medicine: knowledge and skills, attitude, and external factors

**DOI:** 10.1007/s40037-013-0039-2

**Published:** 2013-02-05

**Authors:** Sandra E. Zwolsman, Nynke van Dijk, Ellen te Pas, Margreet Wieringa-de Waard

**Affiliations:** Department of General Practice, Academic Medical Center, PO Box 22700, 1100 DE Amsterdam, the Netherlands

**Keywords:** Evidence-based medicine, General practice, Speciality training, General practice trainees, Barriers

## Abstract

Although efforts are made to integrate evidence-based medicine (EBM) into clinical practice, physicians experience significant barriers to its implementation. The aim of this study is to quantify the barriers that general practice (GP) trainees experience when using EBM in practice. In September 2008, a questionnaire was administered to 140 GP trainees from three Dutch GP Speciality Training Institutes. The questionnaire focused on barriers that GP trainees meet when using EBM in practice. Factor analysis identified components in which barriers exist, and the validity and reliability of the questionnaire were established. After removing four items that did not fit the questionnaire structure, factor analysis identified three relevant components. All three components had similar mean scores, indicating a similar negative influence of these components on the practice of EBM: knowledge/skills (α = 0.72, mean score 2.9 ± 0.8), attitude (α = 0.70, mean score 2.9 ± 0.6), and external factors (α = 0.66, mean score 3.0 ± 0.5). The barrier that trainees experienced most was lack of time to practise EBM. Barriers to the use of EBM were present in three components: knowledge/skills, attitude, and external factors.

## Introduction

**Fig. 1:**
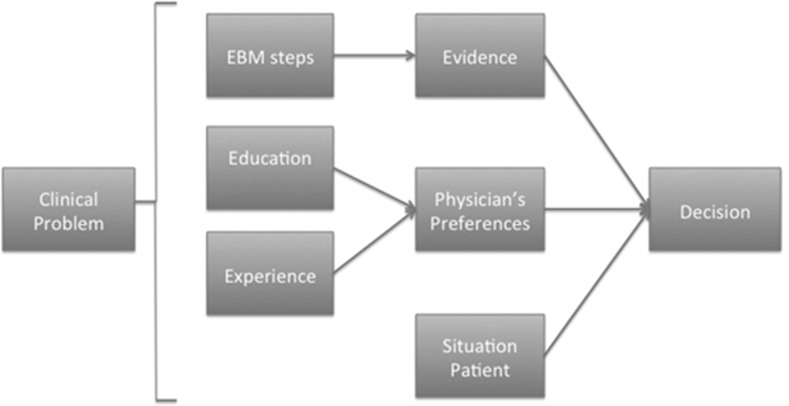
EBM GP trainees

Evidence-based medicine (EBM) was introduced in 1992 [1]. Since then, EBM education has become part of almost all medical education curricula [2]. Formal EBM education usually focuses on the ability of trainees to use the well-known five-step model: ask, access, appraise, apply, and audit [2]. During EBM education, ideally, each step is thoroughly discussed and the translation of the critically appraised evidence into clinical practice is taught [2] (Fig. 1).

However, the transfer of evidence into practice is not optimal [3] and barriers limiting the use of EBM are met by both trainees [4] and their clinical trainers [5]. In order to optimize the transfer of evidence into patient care—through installing EBM education in practice [6]—current barriers need to be tackled. An overview of the barriers that are encountered in the practice of EBM by general practice (GP) trainees could help to define not only which barriers are present, but could also help to determine the relative importance of these barriers, allowing trainers to focus on specific components hindering the use of EBM.

With better knowledge of the barriers that hinder the implementation of EBM, educational and practical tools that focus on overcoming these barriers can be developed. Therefore, the aim of this study is to determine which barriers prevent the smooth implementation of EBM use by GP trainees. Since no validated assessment tool for EBM behaviour—and more specifically for assessing barriers in the use of EBM—exists [7], in this study we describe the development and validation of an instrument that assesses these barriers.

## Method

### Setting

In the Netherlands, GP Speciality Training is a 3 years competency-based training programme with both tutorials (1 day a week) and training in clinical practice (4 days a week). Training takes place in GP practices in the first and third year of the GP Speciality Training Programme; in the second-year, GP trainees are enrolled in 3-or 6-monthly clinical rotations, such as psychiatry, care for the elderly/nursing home, or the emergency department.

### Subjects

In September 2008, first-year GP trainees at the Leiden University Medical Center, the University Medical Center Groningen and the Academic Medical Center-University of Amsterdam (AMC-UvA) were included in this study to determine the barriers to EBM that trainees experience in the first month of starting their training programme.

### Ethical considerations

The questionnaire was simultaneously administered under examination conditions to all trainees at each institute. Written informed consent was obtained from each participant. The study was approved by the Heads of the GP Speciality Training Programmes and performed in accordance with the principles of the Declaration of Helsinki.

### EBM barriers questionnaire

All barriers—identified by a systematic review of barriers faced by the trainees when applying EBM [4]—were formulated as statements with alternately positive or negative phrasing to avoid a ‘response set’ phenomenon [8]. The questionnaire consisted of 19 statements concerning EBM. Answers were given on a 5-point Likert scale (1 = highly disagree, 5 = highly agree).

### Validity and reliability of the questionnaire

The validity and reliability of the questionnaire were assessed. In order to determine how the questionnaire would be received, we presented the questionnaire to a panel of four physicians and four EBM experts and asked whether they thought the questionnaire was suitable for surveying EBM barriers in GP trainees. After the trainees filled in the questionnaire, an exploratory factor analysis was done. Validity analysis showed that four items did not fit the model and therefore these items were removed from the questionnaire. Validity analysis of the new questionnaire—including 15 items—was done using an Oblimin rotation to identify the components in which the barriers are present. In factor analysis, a Kaiser–Meyer–Olkin measure of >0.7, and a Bartlett measure of <0.01 are considered sufficient [9]. We assigned items with factor loadings >0.4 to the related component. For items with factor loadings between 0.3 and 0.4, the contents were judged to determine whether the items fit the component. The reliability (internal consistency) of the questionnaire was determined to test whether the questionnaire assessed the EBM barriers consistently [10]. In order to calculate the internal consistency of the components that were found in factor analysis, the outcomes of items with a negative correlation in the factor analysis were inversed—a Cronbach’s alpha >0.7 was considered satisfactory [10]. The floor and ceiling effects of the outcomes of the components were calculated to see if all scales were sensitive to measuring change—if >15 % of the respondents had the highest or lowest possible score the scale characteristics were found to be satisfactory [10].

The subscales that were identified in the factor analysis were compared with other scales that were assessed in the same study sample (published elsewhere) [11]. The following scales were used as a comparison:

- The McColl questionnaire primarily measures attitude towards EBM. The outcomes of the McColl questionnaire (mean score between 0 and 10; 0 = negative attitude, 10 = positive attitude) and also the trainee’s self-rated attitude (5-point scale; 1 = negative attitude, 5 = positive attitude) were related to barriers in the component ‘attitude’ [12].
- The Berlin questionnaire measures knowledge and skills in EBM. The outcomes of the Berlin questionnaire (mean score between 0 and 10; 0 = negative knowledge, 10 = positive knowledge) and also the trainee’s self-rated knowledge (5-point scale; 1 = no knowledge, 5 = positive knowledge) were related to barriers in the component ‘knowledge/skill’s [13].
- The personal characteristics of the trainees, i.e. years of research experience and years of practice experience, were related to barriers in the component ‘external factors’.

### Analysis of the outcomes

All data were analyzed using SPSS version 16.0. We described the data with proportions for categorical data, with means and standard deviations for normally distributed data, and with median and quartiles for non-normally distributed data. We compared the components with related scales using Pearson’s correlation coefficient for normally distributed data and Spearman’s rho for non-normally distributed data: a coefficient of >0.2 was considered satisfactory [9]. The scores of the three components were compared using a paired samples *t* test.

## Results

### Characteristics of GP trainees

A total of 140 GP trainees (response rate 97 %) filled in the EBM barriers questionnaire: 73 (52.1 %) from the University of Amsterdam, 35 (25.0 %) from Leiden University and 32 (22.9 %) from the University of Groningen (Table 1). The reason for not filling in the questionnaire for the remaining three GP trainees was absence [11].

**Table 1:** Characteristics of trainees (*n* = 140)

Personal characteristics		
Women	*N*	99 (71 %)
Age	Mean, SD	29.3 ± 3.3
Years graduated	Mean, SD	2.2 ± 2.1
Knowledge		
Knowledge (Berlin)	Mean, SD	6.8 ± 2.4 (score between 0 and 15)
Self-reported knowledge	Median, quartiles	3 (2–3) (score between 1 and 5)
Attitude		
Attitude (McColl)	Mean, SD	62.8 ± 8.8 (score between 0 and 100)
Self-reported attitude	Median, quartiles	4 (4–4) (score between 1 and 5)

### EBM barriers questionnaire

The outcomes of the assessment (*n* = 140) were used to determine validity and reliability measures of the questionnaire. Face validity was positive; all panel members agreed that the questionnaire reflects the barriers that GP trainees experience in their use of EBM and that the questionnaire is suitable for general practice. Exploratory factor analysis showed three components: knowledge/skills, attitude, and external factors. Four items were excluded during factor analysis because of insufficient loading on the components (Table 2); [9]. Convergent validity analysis showed the following correlations: knowledge/skills and the outcomes of the Berlin questionnaire (*r* = 0.09, *p* = 0.30), knowledge/skills and self-rated knowledge (*r* = 0.59, *p* < 0.05), attitude and the outcomes of the McColl questionnaire (*r* = 0.39, *p* < 0.05), attitude and self-rated attitude (*r* = 0.36, *p* < 0.05), external factors and years of research experience (*r* = 0.07, *p* = 0.69), external factors and years of practice experience (*r* = 0.19, *p* < 0.05).

**Table 2:** Factor loadings

Scale items	Factor loading	% Variance	α
Knowledge/skills		12.1	0.72
As a result of inexperience with one (or more) of the EBM steps, I do not succeed at using EBM in practice	0.74		
As a result of a lack of education in using EBM, I am unsure of what using EBM practically means	0.74		
My skills in searching for evidence in databases (i.e. PubMed) are sufficient	−0.80		
When I search for evidence I do not know when to be pleased with the answer found	0.37		
Attitude		26.6	0.70
I am not motivated in working according to the principles of EBM	0.61		
I am not interested in searching for the best evidence	0.67		
The time I have per patient is insufficient to also search for answers to my questions (according to the principles of EBM)	0.50		
I do not search for clinical evidence because I rely on the formal education I received during Speciality Training as supplying me with the right knowledge	0.61		
When I have a clinical question, I take the initiative to search for an evidence-based answer	−0.58		
When busy, searching for clinical evidence is not a priority to me	0.61		
External factors		9.1	0.66
My trainer motivates me to use EBM	0.59		
Formal education stimulates me to use EBM in practice	0.63		
My teacher stimulates me to use EBM	0.77		
There is enough guidance in my training practice to support me in using EBM	0.46		
During consultations, I have sufficient time to work according to the principles of EBM	0.54		
Total % of explained variance		47.8	

The scores on the different scales showed a normal curve: floor and ceiling values were not present. The internal consistency measures (alphas) of the constructs were satisfactory: knowledge/skills (0.72), attitude (0.70), and external factors (0.66).

### Barriers experienced by GP trainees

The three single barriers that were experienced most by GP trainees were all time related: ‘When busy, searching for evidence is not a priority to me’ (mean = 3.8, SD = 0.87), ‘The time I have per patient is insufficient to also search for answers to my questions’ (mean = 3.6, SD = 1.02) and ‘During consultations, I have insufficient time to work according to EBM’ (mean = 3.6, SD = 0.79) (Table 3).

**Table 3:** Single barriers to the use of EBM

Barrier	Mean ± SD
As a result of inexperience with one (or more) of the EBM steps I do not succeed at using EBM in practice	2.9 ± 1.0
As a result of a lack of education in using EBM, I am unsure of what using EBM practically means	2.9 ± 1.0
My trainer motivates me to use EBM	3.1 ± 0.8
I am not motivated in working according to the principles of EBM	2.2 ± 0.9
Formal education stimulates me to use EBM in practice	2.6 ± 0.9
My skills in searching for evidence in databases (i.e. PubMed) are sufficient	3.1 ± 1.0
I am not interested in searching for the best evidence	2.4 ± 0.9
The time I have per patient is insufficient to also search for answers to my questions (according to the principles of EBM)	3.7 ± 1.0
My teacher stimulates me to use EBM	2.7 ± 0.8
There is enough guidance in my training practice to support me in using EBM	3.0 ± 0.8
I do not search for clinical evidence because I rely on the formal education I received during speciality training as supplying me with the right knowledge	2.5 ± 0.9
I have difficulties with understanding the English language when reading articles	1.8 ± 0.8
When I search for evidence I do not know when to be pleased with the answer found	3.1 ± 1.0
When I have a clinical question, I take the initiative to search for an evidence-based answer	3.2 ± 0.9
When busy, searching for clinical evidence is not a priority to me	3.8 ± 0.9
I have a fear of repercussions when confronting my clinical trainer with the latest evidence	1.8 ± 0.7
I forget the clinical questions I wanted to search answers for	2.8 ± 0.9
I use the quickest method to answer clinical questions	2.1 ± 0.6
I do not have enough time to use EBM	3.6 ± 0.8

No difference is found among the average scores of the three components (*p* > 0.05).

## Conclusions

The literature states that the practice of EBM by trainees is limited by barriers that prevent trainees from using EBM [4]. In this study we assess the barriers towards EBM as experienced by GP trainees. Factor analysis divided the barriers into three components: knowledge/skills, attitude, and external factors. All groups of barriers were equally present in trainees.

The identified components closely resemble the social–cognitive learning theory of Bandura [14]. Bandura [14] suggests that personal factors, environmental factors, and behaviour reciprocally influence each other. This leads to the assumption that both personal factors (knowledge/skills and attitude) as well as environmental factors can have a strong negative or positive influence on behaviour. The relationship between the EBM-related barriers and the components of this theory supports the likelihood that behaviour could be improved when personal factors (here:knowledge/skills and attitude) and environmental factors (here:external influences) barriers are limited.

In formal education, attention is paid to the knowledge as well as the attitude of the trainees (cognitive factors) [2]. Several studies have assessed the influence of EBM courses on knowledge, and knowledge usually increases when receiving education [13–16]. Knowledge has been proven to be a barrier in the use of EBM by clinical trainers [5]. The question is whether this barrier derives from a true knowledge deficit or from the GP’s perception that his EBM knowledge is lacking. In GPs, attitude is not necessarily negative [12]. However, attitude, if negative, can have a devastating influence on behaviour [12]. The trainer, who is not always a positive role model regarding EBM, could have a large influence on the attitude of the trainees [5]. The effect of EBM education on the environmental factors (colleagues, role models) has, to our knowledge, not yet been studied.

Mapping all the cognitive and external environmental barriers perceived by GP trainees highlights the current state of resistance in using EBM and consequently shows where tools are needed to overcome the barriers. In the clinical training setting of the GP trainee, one major barrier needs to be resolved: trainees and trainers work one-on-one for about a year. If trainers motivate their trainees, trainees may consider the environment they work in less as a barrier. As far as the environment of these trainees is concerned, trainers as role models are important components of the educational environment of trainees. Thereby, they can have an important influence on the attitude of trainees [17]. Besides setting a good example, trainers should focus on EBM training in practice instead of training in the classroom [18]. In practice they have to integrate the evidence, with their experience and the specific situation of the patient [19]. Practice-related barriers, such as access to evidence, influence of colleagues, and practice policy, have a noteworthy influence on EBM use of trainees [4, 20].

There are some limitations to the execution of this study. At first, the barriers in the questionnaire were derived from a review that covered all the healthcare professions [4]. As a consequence, barriers experienced in secondary care were also included in the review and in our questionnaire. However, the barriers that were found in this review were also found in a review that described barriers experienced by GPs [20] and they were recognized by a large number of trainees in our study. We can therefore conclude that no barriers were included in the questionnaire that exclusively account for professionals working within secondary care. Secondly, the educational setting as well as the way in which EBM is taught in the GP speciality training institute may vary from other speciality training programmes. As a result, the outcomes of this study cannot be directly generalized to other educational settings or healthcare disciplines.

### Further research

The translation of knowledge into the clinic is obstructed [3, 21]. Coomarasamy [18] confirms this finding in a review in which he states that education only improves knowledge and that teaching of EBM should foster attitudes and encourage practice. This study shows that barriers to the use of EBM exist in all three components. In order to eliminate barriers regarding the use of EBM—and thereby overcoming problems with unsuccessful implementation of EBM—it is necessary to pay more attention to minimizing the barriers regarding external factors. In education, trainers should be instructed to show the relevance of EBM to the trainees and to teach the trainees how to use EBM in clinical practice. In practice, this means that GP clinical trainers need to be educated in EBM knowledge/skills in order to improve their attitude towards EBM [5]. Also, they need to be specifically taught how to use EBM in practice and should be taught how to eliminate all external barriers in their GP practice [5]. To do so, the formal education that trainees receive at their institute of speciality training should be adapted to improve the implementation of EBM [22], and formal EBM education should be synchronized with the EBM education in clinical practice. Therefore future studies should aim at developing and evaluating educational tools.
